# Oscillatory properties of class C notifiable infectious diseases in China from 2009 to 2021

**DOI:** 10.3389/fpubh.2022.903025

**Published:** 2022-08-11

**Authors:** Yanxiang Cao, Meijia Li, Naem Haihambo, Yuyao Zhu, Yimeng Zeng, Jianhua Jin, Jinyi Qiu, Zhirui Li, Jiaxin Liu, Jiayi Teng, Sixiao Li, Yanan Zhao, Xixi Zhao, Xuemei Wang, Yaqiong Li, Xiaoyang Feng, Chuanliang Han

**Affiliations:** ^1^The National Clinical Research Center for Mental Disorders & Beijing Key Laboratory of Mental Disorders, Beijing Anding Hospital, Capital Medical University, Beijing, China; ^2^Advanced Innovation Center for Human Brain Protection, Capital Medical University, Beijing, China; ^3^Faculty of Psychology and Center for Neuroscience, Vrije Universiteit Brussel, Brussels, Belgium; ^4^College of Environmental Sciences and Engineering, Peking University, Beijing, China; ^5^State Key Laboratory of Cognitive Neuroscience and Learning & IDG/McGovern Institute for Brain Research, Beijing Normal University, Beijing, China; ^6^Zhongshan School of Medicine, Sun Yat-sen University, Guangzhou, China; ^7^School of Artificial Intelligence, Beijing Normal University, Beijing, China; ^8^Baoding First Central Hospital, Baoding, China; ^9^Department of Psychology, University of Washington, Washington, SA, United States; ^10^School of Psychology, Philosophy and Language Science, University of Edinburgh, Edinburgh, United Kingdom; ^11^Faculty of Arts, Humanities and Cultures, School of Music, University of Leeds, Leeds, United Kingdom; ^12^China Academy of Chinese Medical Sciences, Institute of Acupuncture and Moxibustion, Beijing, China; ^13^Institute of Mental Health, Peking University Sixth Hospital, Beijing, China; ^14^Shenzhen Key Laboratory of Neuropsychiatric Modulation and Collaborative Innovation Center for Brain Science, Guangdong Provincial Key Laboratory of Brain Connectome and Behavior, CAS Center for Excellence in Brain Science and Intelligence Technology, Chinese Academy of Sciences, Brain Cognition and Brain Disease Institute, Shenzhen Institute of Advanced Technology, Shenzhen–Hong Kong Institute of Brain Science, Shenzhen Fundamental Research Institutions, Shenzhen, China

**Keywords:** infectious disease, class C, selectivity, recrudescence, China

## Abstract

**Background:**

Epidemics of infectious diseases have a great negative impact on people's daily life. How it changes over time and what kind of laws it obeys are important questions that researchers are always interested in. Among the characteristics of infectious diseases, the phenomenon of recrudescence is undoubtedly of great concern. Understanding the mechanisms of the outbreak cycle of infectious diseases could be conducive for public health policies to the government.

**Method:**

In this study, we collected time-series data for nine class C notifiable infectious diseases from 2009 to 2021 using public datasets from the National Health Commission of China. Oscillatory power of each infectious disease was captured using the method of the power spectrum analysis.

**Results:**

We found that all the nine class C diseases have strong oscillations, which could be divided into three categories according to their oscillatory frequencies each year. Then, we calculated the oscillation power and the average number of infected cases of all nine diseases in the first 6 years (2009–2015) and the next 6 years (2015–2021) since the update of the surveillance system. The change of oscillation power is positively correlated to the change in the number of infected cases. Moreover, the diseases that break out in summer are more selective than those in winter.

**Conclusion:**

Our results enable us to better understand the oscillation characteristics of class C infectious diseases and provide guidance and suggestions for the government's prevention and control policies.

## Introduction

The epidemic of infectious diseases has a significant impact on all aspects of people's lives, such as people's daily activities (1, 2), education (3–6), diet (1, 7), mental health (8–13), and even countries' economies and development (14, 15). Three types of infectious diseases (class A, B, and C) were defined by the Chinese government using the country's infectious disease surveillance system after the epidemic of Severe Acute Respiratory Syndrome (SARS) in 2003 (16). The large-scale epidemic in China is mainly caused by class B and class C infectious diseases, since the rate of infection of class A notifiable diseases such as the plague and cholera has been controlled at a very low level in China. Besides, the mechanisms of the class B disease epidemic have been well systematically studied in both temporal and (17, 18) and spatial scales (19). However, although it is more likely for ordinary people to be infected with class C infectious diseases (such as influenza), very few studies tried to systematically understand the class C infectious diseases together in the mainland of China (20), especially from the oscillatory perspective.

Recrudescence of infectious disease causes the oscillatory phenomenon of epidemics (21). This periodic recurrence has been a common feature (22–24) of infectious diseases around the world (25–34). Currently, the oscillatory properties of infectious diseases are thought to be driven by two main factors: natural and human factors. The natural factors include the seasonal temperature (21, 35), rainfall (21, 36) and natural disasters (37), and the human factors include the school terms (38, 39), economic migration (40, 41), vaccination coverage (42), habitat disruption (43), or some other government epidemic prevention policies (21, 44). Understanding the oscillatory properties of the infectious disease could provide essential information for the forecast and avoid economic loss and harm to the people's health (8, 45–47).

Therefore, in the present study, we aimed to fill the gap of insufficient studies in the oscillatory property of class C diseases. To be more specific, we attempted to categorize the diseases based on their oscillatory frequencies; then, we investigated the relationship between the oscillation strength and the number of infected cases; based on this, we narrowed the time scale to 1 year and inspected the seasonal selectivity. We collected time-series data for the monthly reported confirmed cases of nine class C notifiable infectious diseases from 2009 to 2021 and calculated their power spectrums by multi-taper methods. We also calculated the oscillation power and the average number of infected cases of all nine diseases in the first 6 years (2009–2015) and the next 6 years (2015–2021) and their correlation. In addition, we conducted the correlation analysis between the preferred month and the selectivity of the infectious diseases.

## Materials and methods

### Data and sources

Available time-series data for the monthly reported and confirmed cases of nine class C notifiable infectious diseases in China's mainland, from June 2009 to September 2021, was obtained from the National Health Commission of China (http://www.nhc.gov.cn/). The monthly dataset is open to the public, reported by the Chinese Center for Disease Control and Prevention (CDC) (See Supplementary materials). These diseases are Flu (Influenza), Mumps, Rubella (German measles), Acute hemorrhagic conjunctivitis (Apollo disease), Leprosy (Hansen's disease), Scrub Typhus (Bush typhus), Leishmaniasis (Black fever, or Kala-Azar), Echinococcosis (Hydatid disease), and Hand, foot, and mouth disease (Table 1). The data sampling rate is number of cases per month (12 time points per year) by the monthly report of the National Health Commission of China.

**Table 1:** Summary of the main finding in nine class C infectious diseases in mainland China.

**Name of infectious diseases**	**Type**	**Preferred month**	**Selectivity**	**Changes in the number of infected cases from first 6 years to second 6 years**	**Changes in the power of infected cases from first 6 years to second 6 years (log)**
Flu (Influenza)	1	Jan	0.79	0.22	0.03
Mumps	2	Jun	0.65	1.58	4.07
Rubella (German measles)	1	May	0.88	3.92	6.00
Acute hemorrhagic conjunctivitis (Apollo disease)	1	Sept	0.62	0.98	2.77
Leprosy (Hansen's disease)	1	Mar	0.50	1.66	3.81
Scrub Typhus (Bush typhus)	1	Oct	0.71	1.90	2.39
Leishmaniasis (Black fever, or Kala-Azar)	1	Dec	0.35	1.18	1.11
Echinococcosis (Hydatid disease)	3	Dec	0.47	0.81	0.82
Hand, foot and mouth disease	1	Jun	0.92	1.06	1.39

### Spectrum analysis

The spectrum analysis was used to better quantify the oscillatory property of each infectious disease. Similar methods have been used in classic and modern studies in the field of infectious diseases (23, 25, 27, 48, 49). Spectrum analysis is a technique for decomposing complex signals into simpler signals based on the Fourier transform. Most biological signals could be expressed as the summation of the various simple signals of different frequencies and produce information of a signal at different frequencies (such as amplitude, power, intensity, or phase, etc.) (50–53).

**Figure 1:**
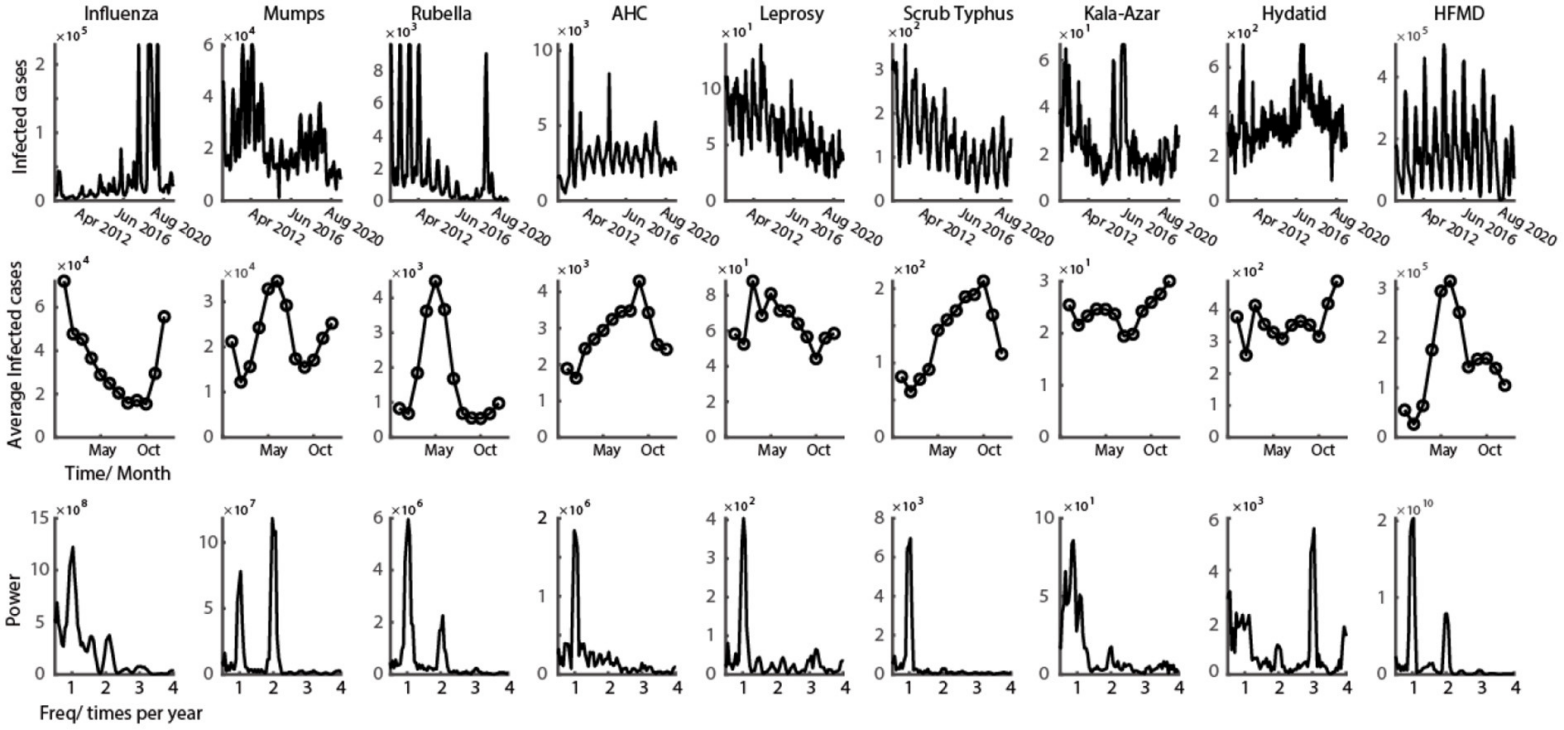
Representative class C infectious disease with clear oscillation pattern. First row shows the time series of monthly infected cases from 2009 to 2021 for nine class C infectious diseases. Second row shows the average number of infected cases every month in a year. Third row illustrates the power spectrum calculated from the data of first row (data in the figure could be found in Supplementary materials).

The power spectral density (PSD) for each infectious disease during these 12 years was computed using the multi-taper method using the Chronux toolbox (54) [an open-source, data analysis toolbox (Chronux) available at http://chronux.org]. The multi-taper method attempts to reduce the variance of spectral estimates by pre-multiplying the data with several orthogonal tapers, yield a more reliable ensemble estimate of noisy data. Power spectra of the time-series data of infected cases of each disease was calculated from 2009 to 2021 (Figure 1 graphs on the bottom), which has been used in the earlier work of our lab (2, 17).

### Classification of different clusters of diseases

We used two oscillatory features to classify the different clusters of infectious diseases: the power ratio (Ratio 1) between once a year and twice a year, and the power ratio (Ratio 2) between once a year and three times a year. The definition of power ratio is the ratio between the powers corresponding to two different frequencies (times per year). We then set two linear thresholds (Ratio 1 = 1; Ratio 2 = 1) that precisely separated them into three clusters.

### Tuning curves for monthly infected cases

The tuning curve was computed by taking the monthly average number of infected cases- during all 12 years, which is under the assumption that all infectious diseases included in this study have a similar trend each year.

### Preferred month and selectivity of the epidemic outbreak

Two indices were defined to better capture the property of oscillations for infectious diseases in a year: preferred month and infection selectivity. The preferred month is defined as the month in a year that has the most cases of infections. The infection selectivity index is defined as 1 minus the ratio of the minimum and the maximum number of infected cases in a year. If the selectivity index is closer to 1, then the shape of the tuning curve is sharper, and vice versa.


$$\begin{array}{l} selectivity_{index}=1-\frac{\min\left(mean\;infected\;cases\;in\;a\;year\right)}{\max\left(mean\;infected\;cases\;in\;a\;year\right)} \end{array}$$

### Correlation analysis

We used the Pearson correlation to measure the relationship between the change in infected cases and the change in oscillation power of the infectious diseases on all nine infectious diseases. The Pearson correlation was also used in the correlation analysis between the selectivity index and the preferred month.

## Results

The oscillatory patterns in infectious diseases' time series in mainland China is clear over the past 12 years (Figure 1, graphs on the top). According to the 12-year historical data of class C infectious diseases, six out of nine diseases showed a diseasing trend of the infectious number of people, and three of them still increased (Table 1). From the view of the change of the infection oscillation, seven out of nine diseases showed a diseasing trend (Table 1). The sampling rate of the data for each disease is 12 data points per year, with one data point representing 1 month.

### Three clusters of the oscillatory patterns of the class C infectious diseases

**Figure 2:**
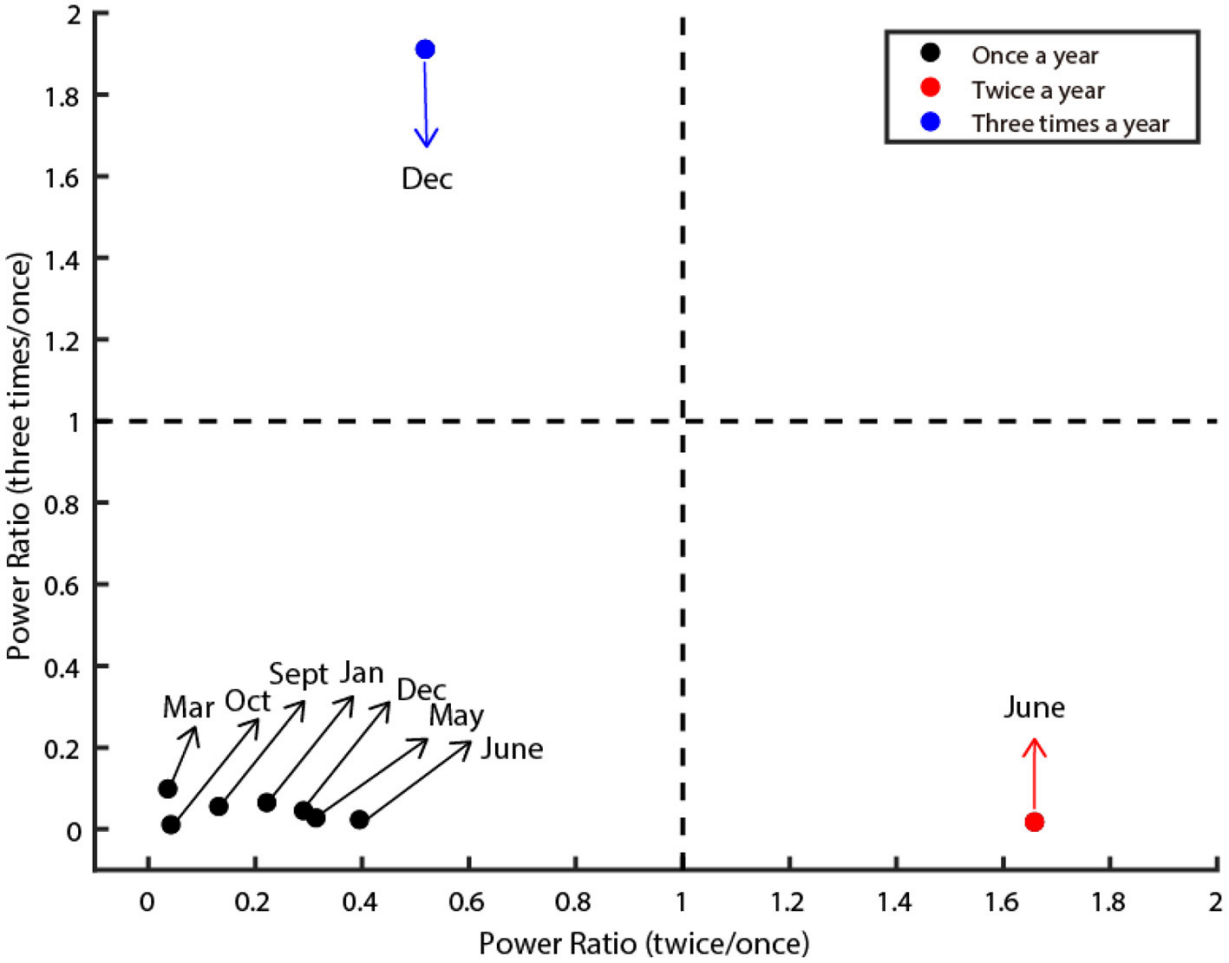
Three oscillatory types of class C infectious diseases. The figure illustrates three clusters (denoted by red, black, and blue dots). The X-axis denotes the power ratio of occurrence between twice a year and once a year. The Y-axis denotes the power ratio of occurrence between three times a year and once a year. The dashed line is the criteria that separates them. The dots in the lower left depict diseases classified as Type I. The dots in the lower right corner depict diseases classified as Type II. The dots in the upper left corner are classified as Type III. The preferred month of each disease was marked by the arrow.

The time-series data of nine class C infectious diseases were shown in the first row of Figure 1. It is obvious that the epidemic of all nine diseases shows a strong oscillatory phenomenon. To better interpret the periodic properties throughout a year, the average of all 12 years' data was calculated (number of infected cases are represented in the second row of Figure 2). From the infection tuning curve of each disease, they have different preferred months to outbreak. Different infectious diseases also have different numbers of outbreak peaks during 1 year, which suggests the frequency of the outbreak of an epidemic disease in a year. We then calculated the power spectrum of the nine diseases using 12 years' time series data. Each infectious disease in this study has a tuning curve, and the oscillatory pattern within a year is clear. As predicted by the tuning curve (second row of Figure 1), multiple frequency peaks were shown in the spectrum.

Next, we quantified the oscillatory properties of different diseases, and found they could be classified as three distinct clusters, which are illustrated in Figure 2. The horizontal axis of this panel denotes the power ratio between twice a year and once a year, and the vertical axis denotes the power ratio between three times a year and once a year. The larger the value of the horizontal axis is, the more probable the oscillation is twice a year. The larger the value of the vertical axis is, the more probable the oscillation is three times a year. Then we set two thresholds that precisely separated them into three clusters (dashed line in Figure 2). In total, seven out of nine diseases belong to Type I (Influenza, Rubella, AHC, Leprosy, Scrub Typhus, Kala-Azar, and HFMD), one out of nine diseases belong to Type II (Mumps), the remaining one disease (Hydatid) belongs to Type III (Figure 2).

### Positive correlation between the change of infected cases and change of oscillatory power

**Figure 3:**
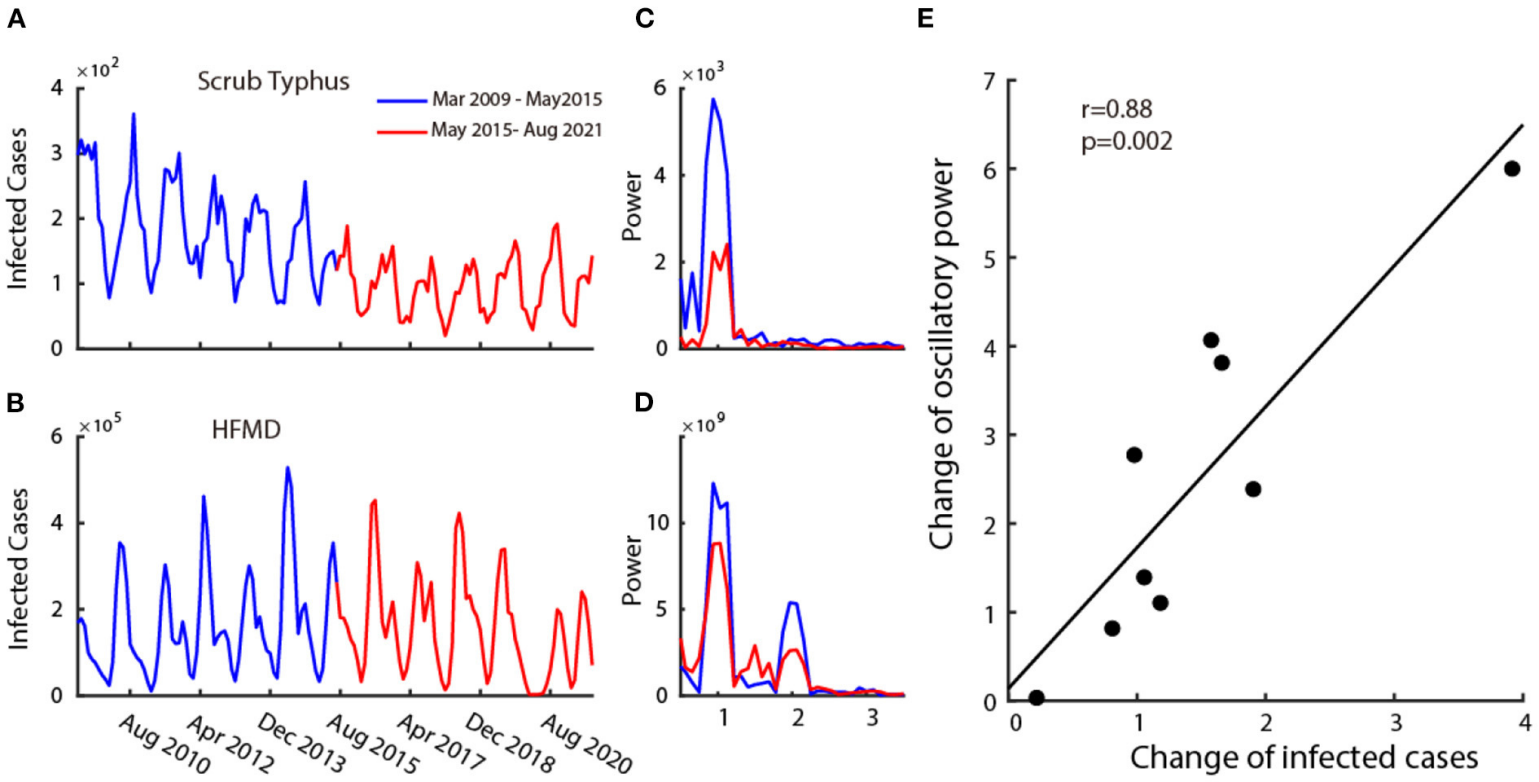
Relationship between the infection and its oscillatory strength. Plot **(A,B)** are two examples of disease's time series monthly infected cases from 2009 to 2021. The blue curve shows the time series of the first 6 years (2009–2015) and the red curve shows the time series of the last 6 years (2015–2021). Plot **(C,D)** show the power spectrum calculated in the first 6 years (blue curve) and the last 6 years (red curve) corresponding to the time-series data of **(A,B)**. Plot **(E)** shows the scatter plot of change in mean infected cases and change in oscillatory power.

Next, we split the 12-year dataset into two parts: the first 6 years (2009–2015) and the last 6 years (2015–2021). In these 12 years, the number of infected cases of six (Mumps, Rubella, Leprosy, Scrub Typhus, Leishmaniasis, HFMD) out of nine infectious diseases decreased over time (Figure 3A for a typical example), and others (Flu, AHC, and Echinococcosis) remained unchanged or increased (Figure 3A for a typical example of the unchanged case). This information is summarized in the 5th column of Table 1.

We then explored the relationship between the change in the number of infected cases and the corresponding strength of oscillatory power. To this end, we calculated the power spectrums in two time periods (2009–2015 and 2015–2021) for all nine infectious diseases. The change in the number of infected cases is defined as the ratio of the mean infected cases each month between 2009 and 2015 (Figures 3A,B, blue curve) and 2015–2021 (Figures 3A,B, red curve), and the change in the oscillatory power is defined as the ratio of the average power spectrum between 2009 and 2015 (Figures 3C,D blue curve) and 2015–2021 (Figures 3C,D red curve). We then performed a correlation analysis between the change in infected cases and the change in oscillation power of the infectious diseases on all nine class C infectious diseases. We found that there is a strong positive correlation (Figure 3E) (*r* = 0.88, *p* = 0.002, Pearson correlation). This illustrates that the increase in oscillation strength often accompanies the increase in the number of infected cases.

## Discussion

In this study, we systematically explored the oscillatory properties of the nine class C infectious disease in the mainland of China. All the nine class C diseases were found to have strong oscillations, which could be divided into three categories according to their oscillatory frequencies each year. We also found a strong positive correlation between the change of oscillation power and the change in the number of infected cases.

### Comparison with previous works

To our knowledge, this is the first work to directly investigate the oscillatory properties of the nine class C infectious diseases together in mainland China. Previous studies mostly targeted one specific disease [Influenza (55), Mumps (56–60), Rubella (61–63), AHC (64–68), Leprosy (69–72), Typhus (73–76), Leishmaniasis (77, 78), Hydatid disease (79–81), HFMD (82–86)], more in a statistical description sense on the numbers, ratios, or dissecting multiple components to fit the data. These studies seldom investigated these diseases directly from the oscillatory view, but we focused more on the ubiquitous oscillatory property of these class C diseases together to find some common laws as a whole. The prior work on the oscillatory property of the class B diseases (17) has shown that the oscillatory phenomenon is widely found. Similar to the results in this work, we also found that the oscillation is universal in class C infectious diseases.

### Multiple types of the class C infectious diseases

Another issue is the classification of the class C infectious diseases. Generally, people distinguish them by the transmission mode (through respiratory tract, digestive tract, blood sucking insects, and contact transmission). It is noticeable that we proposed a new method to make the clusters through the oscillatory frequency of each infectious disease. Clearly, we found that three clusters were dependent on their outbreak frequencies in a year, which is also consistent with the results in the class B diseases (17). This new classification method could help us better understand the epidemical features on their periodic properties. We also found that most of the diseases (including both class B and C diseases) belong to the first type, which means it outbreak once a year, and the proportion of Type II and III diseases is relatively smaller. The specific mechanism to illustrate their significant difference should be a question that to be answered in the future work.

### Relationship between selectivity and the time of outbreak preference of the infectious diseases

The tuning curves of the infectious diseases were always shown up in previous research (20, 28, 30), but the relationship between the infection selectivity and the preferred outbreak month of the infectious diseases were seldom analyzed. The higher selectivity of the disease means that it only outbreak in some specific month but rarely outbreak at other times.

### Current trend of class C infectious diseases in China

Majority of the class C diseases showed a diseasing trend of the infectious number of people and change of the infection oscillation (Table 1). Combining the results of the change of infection and its oscillation, we could draw conclusions that the infection number is always accompanied by its oscillatory strength for the class C diseases (class B diseases also obeyed with this law), which is consistent with the hybrid model (17). Besides, the oscillatory properties (frequency, preferred month) of a disease remains similar, hence the cluster would be stable using the current method.

Our results precisely describe the oscillatory properties of class C infectious diseases in China, which might help us better understand their fluctuation characteristics, and provide guidance and suggestions for government prevention and control policies. From the oscillatory view to recheck the information related to the infectious diseases helps us better understand the time and extent of their outbreak. In the future, the number of people infected with all infectious diseases might not decrease to zero, but the ultimate goal of prevention is to minimize as much as possible the losses caused by the infectious diseases.

## Data availability statement

The original contributions presented in the study are included in the article/Supplementary materials, further inquiries can be directed to the corresponding author.

## Author contributions

CH, YC, and ML conceived and designed the study. CH, ML, NH, YZe, JJ, and ZL contributed to the literature search. CH, JL, JT, SL, YZhu, YZha, XZ, XW, and YL contributed to data collection. CH, YC, XF, and JQ contributed to the data analysis and the interpretation of results. All authors contributed to writing the paper.

## Funding

This work was funded by the Beijing Municipal Hospital Clinical Technology Innovation and Research Plan (XMLX201805), Beijing Municipal Hospital Research and Development Project (PX2021068), Advanced Innovation Center for Human Brain Protection Project (3500-12020137), and Beijing An Ding Hospital, Capital Medical University (YG2021-06).

## Conflict of interest

The authors declare that the research was conducted in the absence of any commercial or financial relationships that could be construed as a potential conflict of interest.

## Publisher's note

All claims expressed in this article are solely those of the authors and do not necessarily represent those of their affiliated organizations, or those of the publisher, the editors and the reviewers. Any product that may be evaluated in this article, or claim that may be made by its manufacturer, is not guaranteed or endorsed by the publisher.
